# Preliminary Examination of the Biological and Industry Constraints on the Structure and Pattern of Thoroughbred Racing in New Zealand over Thirteen Seasons: 2005/06–2017/18

**DOI:** 10.3390/ani11102807

**Published:** 2021-09-27

**Authors:** Kylie A. Legg, Erica K. Gee, Darryl J. Cochrane, Chris W. Rogers

**Affiliations:** 1 School of Veterinary Science, Massey University, Private Bag 11-222, Palmerston North 4442, New Zealand; E.K.Gee@massey.ac.nz (E.K.G.); C.W.Rogers@massey.ac.nz (C.W.R.); 2 School of Sport, Exercise and Nutrition, Massey University, Private Bag 11-222, Palmerston North 4442, New Zealand; D.Cochrane@massey.ac.nz; 3 School of Agriculture and Environment, Massey University, Private Bag 11-222, Palmerston North 4442, New Zealand

**Keywords:** horse, Thoroughbred, racing, injury, system dynamics

## Abstract

**Simple Summary:**

The training and racing schedules of Thoroughbred racehorses vary within a racing jurisdiction. Changes in regulation at a local or national level can alter the frequency of racing, age profile and the number of and duration of spells (rest periods) for horses in active race training. In order to consider any intervention to reduce injury risk at an industry level, it is important to understand all the parameters pertinent to the racing jurisdiction; both those constrained by biology and by the economics of the racing system. The number of horses and races in New Zealand is decreasing, with a concurrent increase in both the age of horses and the proportion of fillies racing. However, the pattern of race starts remained the same, highlighting the individual biological constraints of the horses in the system. If the industry is considered as a bio-economic system, then the racing system will converge towards efficiency and optimisation of resources. Within this framework, the described trend is likely to continue if the number of horses entering the system continues to decrease. Therefore, these data present a basis for the modelling of changes in racing regulations and injury intervention strategies within this conceptual framework to account for the economic and biological constraints.

**Abstract:**

This study aimed to examine thirteen seasons of flat racing starts (*n* = 388,964) in the context of an ecological system and identify metrics that describe the inherent characteristics and constraints of the New Zealand Thoroughbred racing industry. During the thirteen years examined, there was a 2–3% per year reduction in the number of races, starts and number of horses. There was a significant shift in the racing population with a greater number of fillies (aged 2–4 years) having a race start, and subsequent longer racing careers due to the inclusion of one more racing preparation post 2008 (*p* < 0.05). Additionally, there was an increasingly ageing population of racehorses. These changes resulted in more race starts in a career, but possibly because of biological constraints, there was no change in the number of race starts per season, starts per preparation, or days spelling between preparations (*p* < 0.05). There was no change in the proportion of horses having just one race start (14% of new entrants), indicating that the screening for suitability for a racing career remained consistent. These data identify key industry parameters which provide a basis for future modelling of intervention strategies to improve economic performance and reduce horse injury. Consideration of the racing industry as a bio-economic or ecological model provides framework to test how the industry may respond to intervention strategies and signal where changes in system dynamics may alter existing risk factors for injury.

## 1. Introduction

Thoroughbred racing is a large international industry where uniformity in the rules and regulation of the industry are achieved through the International Federation of Horse Racing Authorities. Despite restriction to the use of a single breed of horse (Thoroughbred) and international racing regulation rules, there are documented differences in the phenotype and genotype of the horses and the organisation of the industry at a national level [1]. These biological and economic differences highlight the potential limitation in extrapolating, or generalising findings from one racing jurisdiction and directly applying these to another jurisdiction.

Musculoskeletal injury (MSI) is the most common reason for involuntary loss for the horse racing industry, accounting for 80% of involuntary interruptions to training and 25% of horses exiting from the industry [2,3]. MSI’s are commonly due to bone fatigue, a function of the number of cycles (strides) and the magnitude of the load applied to the limb [4,5,6]. Both increased and decreased cumulative distance of high-speed training exercise have been presented as a risk factor for musculoskeletal injury, indicating a nonlinear relationship with risk [7,8,9,10,11]. In a recent study, more than half of Australian Thoroughbred training programmes examined exceed previously reported risk levels for MSI with high volumes of gallop work [6,12], but their fracture risk (in races) is lower than reported in other jurisdictions [13]. The complex relationship between training intensity, speed and rest periods [14,15] indicates that the pattern of both training and racing loads may play a large part in injury risk.

The pattern of racing and training are constrained within a jurisdiction by biological (inherent horse and training environment characteristics) and economic drivers, such as availability of prize money and suitable races. If each racing jurisdiction has a unique set of economic and physiological constraints, it is therefore important to consider these jurisdictions in the context of a balanced ecological system, i.e., one which is sustainable. Using this framework, each jurisdiction would inadvertently migrate towards the optimisation of both the economic and physical (biological) resources. This need for the broader consideration of the inter-relationship of variables highlights the opportunity to examine Thoroughbred racing and training practices in the context of bio-economic models [16] or as complex systems [17].

Therefore, the purpose of this study was to examine the New Zealand racing industry in the context of an ecological system and identify metrics that describe the inherent characteristics and constraints of that system. Identification of key industry parameters should provide a basis for future modelling of intervention strategies to improve economic performance and reduce horse injury, allowing comparison between their response with other racing jurisdictions.

## 2. Materials and Methods

Data from all Thoroughbred flat racing starts for thirteen racing seasons (2005/6–2017/18) were supplied by New Zealand Thoroughbred Racing (NZTR), the governing body for Thoroughbred racing in New Zealand. A racing season began on the 1 August and ended on the 31 July. Data were provided at the race start level and the following variables were extracted and used for further analysis: race date, race number, race distance, horse name, age and sex.

A racing meeting was defined as a single day when races were held by one racing club. A race start was defined as a horse entering in a race. A racing preparation (prep) was defined as <60 days between two consecutive races for a horse. If there were ≥60 days between two consecutive races for a unique horse, they were considered to have had a ‘spell’, defined as a period of time where they were not in active race training (rest period). The number of races, days between races within a prep and the length of spell were calculated based on the successive race dates for each unique horse and sorted into a database according to age of horse and racing season.

Racing distances were divided into two categories: ≤1670 m and >1670 m. This distance was selected of being equivalent to a mile and accounts for regional track differences with median racetrack length of 1800 m (interquartile range [IQR] 1600 m–1800 m) [18]. Horses were grouped into two age groups: <6 years old and ≥6 years old. The age distribution of racing horses was determined using Simpson’s Diversity Index using these two groups of horses. As the evenness of racing horses in each group increase, Simpson’s Diversity Index increases.

The integrity of the data was checked using histograms, scatter plots and box plots, where outliers or points of interest were compared with the official NZTR database. Descriptive statistics were used to describe the data at population and seasonal levels. Horses with only one race start were excluded from further analysis of racing profiles. Normality of the data was assessed using an Anderson-Darling test and Kruskal–Wallis tests for significance were used to compare differences between groups. Post hoc tests for significance between groups were assessed with the Chi Squared (χ^2^) test for frequency data and Mann–Whitney U tests for continuous data. Horses were divided into two groups, those that began racing before 2008/9 season, and those that began afterwards. The 2008/9 season was chosen as a cut-off as this was the first New Zealand season after the Global Financial Crisis and there was an associated decline in races, starts and horses after this season. Kaplan–Meier survival curves were used to compare the horse career lengths and number of preps between these two groups. Medians calculated from the data were used to determine optimal values of horses racing. All statistical analyses were conducted using RStudio (version 3.5. 1, 2018; R Foundation for Statistical Computing, Vienna, Austria) with a level of significance set at *p* < 0.05.

## 3. Results

**Figure 1:**
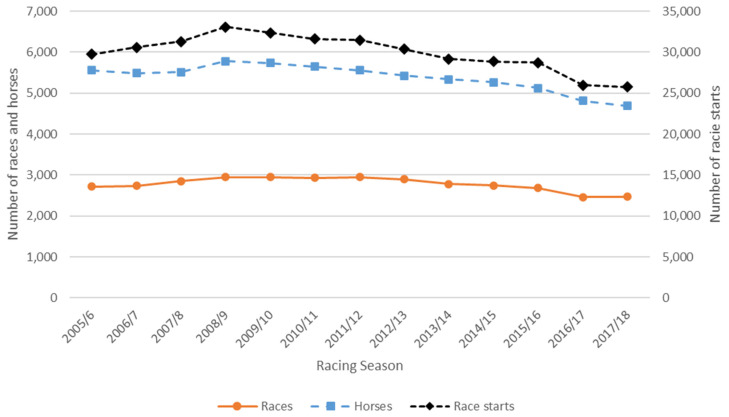
Number of Thoroughbred flat races, horses, and race starts per season for the 2005/6–2017/18 racing seasons in New Zealand.

During the period 1 August 2005–31 July 2018 there were 388,964 flat race starts involving 30,254 unique horses. The number of races, racing starts, and horses peaked during the 2008/9 racing season, afterwards declining at a linear rate of 2–3% per season (Figure 1). For all thirteen seasons, the mean (±SD) number of races per season was 2778 ± 163 and this decreased after the 2008/9 season in a linear relationship at a rate of 59 (2%) races per season (R^2^ = 0.86) (Figure 1). The number of races per racing meeting reduced from a median of 10 (IQR 8–10) in 2005/6–2010/11 seasons to 8 (IQR 7–9) in subsequent seasons, though the number of starters per race was maintained at a median of 11 (IQR 9–13) throughout the period. The mean number of racing starts per season was 30,942 ± 2226 and this decreased after the 2008/9 season in a linear relationship at a rate of 843 (3%) starts per season (R^2^ = 0.96). The mean number of horses who had a race start each season was 5379 ± 320, and this decreased after the 2008/9 season at a rate of 120 (2%) horses per season (R^2^ = 0.95). Number of racing starts had a linear relationship with number of horses racing, with 6.5 starts per horse racing (R^2^ = 0.95).

### 3.1. Pattern of Racing

There were 10 or more starters in 77% of all races (and over 12 starters in 55% of all races) between 2005/6–2017/18, with a maximum number of 18 starters in 2% of races. The median number of starters per race dropped from 11 (IQR 9–13) starters in the 2005/6 season to 10 (IQR 8–12) in the 2017/18 season (R^2^ = 0.29). There were fewer starts in the winter seasons (20%, *p* = 0.02) and an even distribution (26–28%) of starts over the remaining three seasons. The percentage of horses with only one start in their career remained constant at 14.1 ± 0.5% across the thirteen seasons (Table 1). The number of new entrants to racing decreased by 49 (0.4%) horses per year (R^2^ = 0.83). Older horses (≥ 6 years of age) accounted for an increasing percentage of race starts, changing the age profile of the racing population of racehorses. The biological constraints of racing remained stable for the individual horses across the years studied (Table 2). The median number of starts per horse each season was 5 (IQR 2–8) and remained consistent across the thirteen seasons (*p* = 0.1). The median number of days between races within a prep increased by 2 days (from 16 to 18 days) over the thirteen-year period (R^2^ = 0.81).

Between the 2005/6 and 2017/18 racing seasons, the median career length of horses with more than one career start was 451 days (IQR 161–849). The median number of starts over the career of a horse was 9 (IQR 4–19). Horses had a median of 3 (IQR 2–4) racing preps during their career, with a median of 3 (IQR 2–6) races within a prep. The median number of days between races within a prep was 17 (IQR 13–23). The median prep length was 64 days (IQR 34–115) and the median spell length (time between preps) was 161 days (IQR 105–228). Horses had a median of 2 preps per season (IQR 2–2).

**Figure 2:**
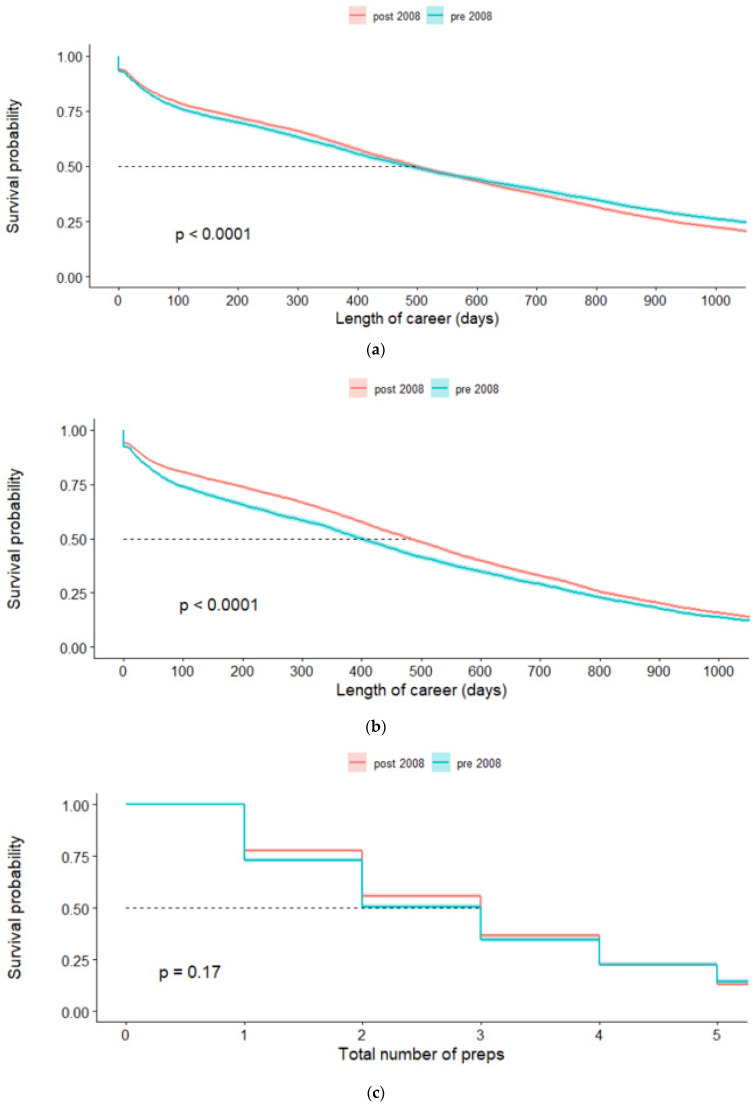

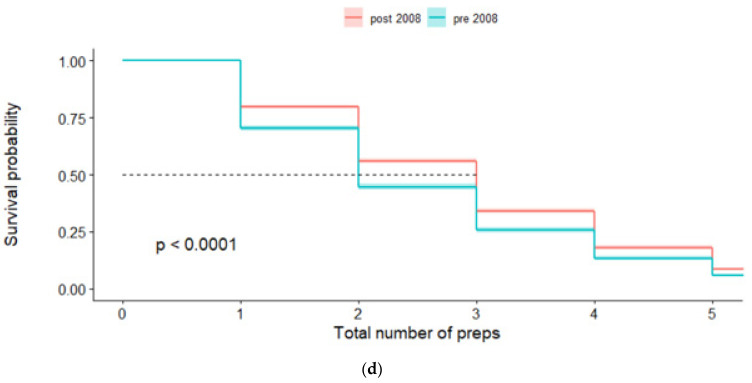
(**a**) Career length for New Zealand flat racing male Thoroughbred horses who had the first race pre-2008 (*n* = 5838) and post-2008 (*n* = 9261). (**b**) Career length for New Zealand flat racing female Thoroughbred horses who had the first race pre-2008 (*n* = 5296) and post-2008 (*n* = 9874). (**c**) Number of preps for New Zealand flat racing male Thoroughbreds who had their first race pre-2008 (*n* = 5838) and post-2008 (*n* = 9261). (**d**) Number of preps for New Zealand flat racing female Thoroughbreds who had their first race pre-2008 (*n* = 5296) and post-2008 (*n* = 9874). Shaded area shows 95% confidence interval.

The career length for mares racing increased post-2008 compared with pre-2008 (Figure 2a,b). Accordingly, more mares remained in racing for an extra prep post-2008 (Figure 2c,d). Horses showed a consistent pattern of racing, with no variation in prep or spell length for horses with increasing number of preps (maximum number of preps for one horse was 14).

### 3.2. Race Distance

The majority (76%) of flat races were run at distances < 1670 m or approximately 1 mile, with little variation in the distances of races offered over the thirteen years (*p* > 0.05). The 24% of races run over a mile (middle-distance) races ranged from 1900–2200 m. Individual horses raced within a narrow range of distances. The median distance change between races for an individual horse was less than one furlong (160 m, IQR 0–200 m). If the horse changed their distance early on in their prep, the race distance was likely to increase rather than decrease.

The median number of days for a horse competing in two subsequent races was 21 (IQR 14–38) for races under a mile and 15 (IQR 11–21) for middle distance races.

### 3.3. Age Profile

Half (51 ± 7%) of the horses had their first race start at 3 years old, and 18 ± 4% of horses had their first race start as a 2-year-old.

The median number of starts per season peaked at 6 starts (IQR 3–10) for 5 and 6-year-old horses, whereas 2-year-old horses had a median of 2 starts per season (IQR 1–3) (Table 3). Two-year-old horses had longer times between races within a prep (*p* < 0.05), but shorter spell lengths than horses ≥ 6 years of age (*p* < 0.05). The percentage of older (≥6 years) horses racing increased by 0.5% a year (R^2^ = 0.7) across the thirteen years from 18% to 24%, whilst the percentage of 2-year-old horses entering races remained constant at 3% of race starters. When the number of starts of horses were divided into two age populations (under and over 6 years), Simpson’s Index increased from 0.30 in 2005/6 racing season to 0.36 in 2017/18 (indicating a growing population of older horses) (Table 1). Younger horses (ages 3–5 years) dominated the shorter distance races, with middle distance races more likely to have older (≥6 years old) horses competing (*p* < 0.05).

**Figure 3:**
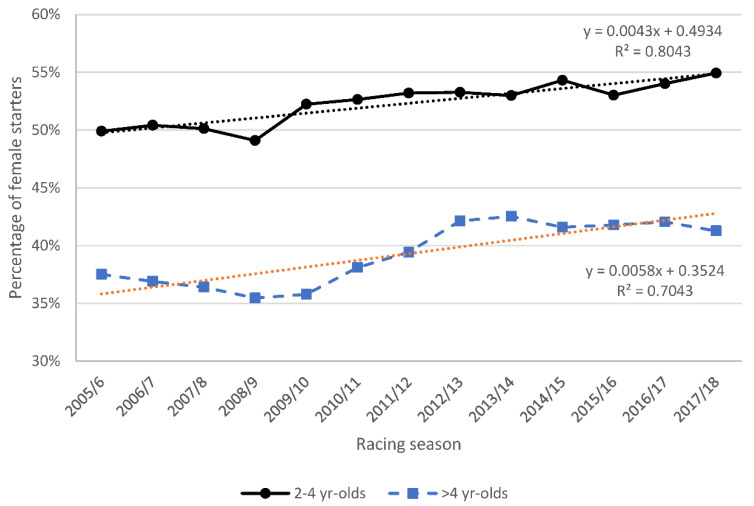
Percentage of female horses (aged 2–4 years and >4 years) racing in the 2005/6–2017/18 racing seasons in New Zealand.

The proportion of 2–4 year-old female horses racing increased linearly by 5% (R^2^ = 0.80) between the 2005/6 and 2017/18 racing seasons (Figure 3). For all years, the proportion of females racing after age four declined by 10% per year in a linear relationship (R^2^ = 0.99). Mares comprised 32 ± 3% of the population of racing horses aged ≥ 6 years, this proportion also increased linearly by 5% (R^2^ = 0.6) over the thirteen years. 

### 3.4. Racing Optimisation

**Figure 4:**
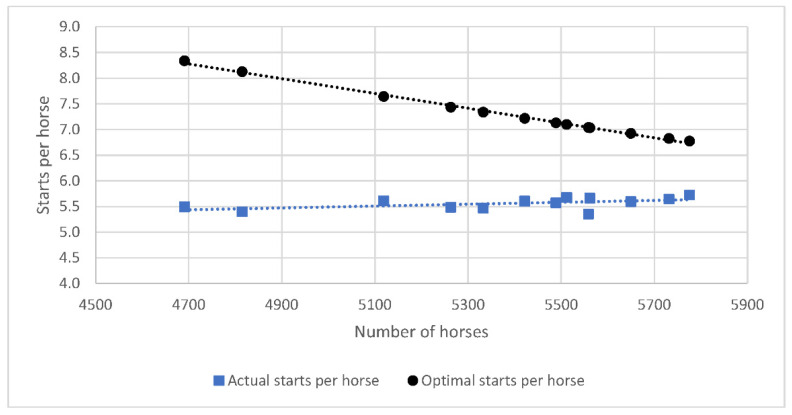
Optimal (threshold) and actual number of starts per Thoroughbred horse racing in the 2005/6–2017/18 racing seasons in New Zealand.

To determine the optimum number of horses required to maintain the current level of race days in New Zealand, it was assumed that 12 starters per race and 10 races per race day were required, as stated in official racing industry publications [19,20] at the current average (±SD) number of race days per year (326 ± 18). Median values to describe the biological constraints of the system (days between races, preparation length, spell length) were taken from the median values calculated above. It was assumed that the number of horses with one start remained constant at 14.1% and the median number of starts for the 7.3% of 2-year-old horses competing each season was restricted to two. The number of extra starts for each horse to optimise racing efficiency per season was related to the number of horses racing in a negative linear relationship (R^2^ = 0.67). Due to the falling number of horses racing, to meet optimal racing efficiency, each horse racing in the 2017/18 season would need to complete 2.5 extra starts, whereas for the 2005/6 season only 1.1 extra start per horse racing was required to meet this threshold (Figure 4). These extra starts could be met by incrementally decreasing the spell length of horses in race training by 2 days for every 100 horses lost from racing (R^2^ = 0.58).

## 4. Discussion

This study provides a comprehensive insight of multiple aspects of the Thoroughbred industry over 13 racing seasons in New Zealand. Summary statistics have previously described the training and racing schedules of specific cohorts of horses involved in the Thoroughbred industry and differences between jurisdictions in both training and racing, MSI and wastage risk have been identified [12,21,22,23]. However, these aspects considered in isolation do not encompass the varying dynamics of the industry as whole.

If the racing industry were treated as a bio-economic model, then the optimisation of returns depends on having both sufficient horses in a race and a maximal number of races per season. The economics drives greater participation and race starts, but the industry is in turn constrained by the biology of the horse and horse welfare. This concept of a bio-economic model, or balanced ecology of the racing industry, is demonstrated by the compensatory mechanisms that have evolved to attempt to optimise the economics of the industry with falling numbers of horses. Since the 2008/9 season, horses have longer careers, staying in for an extra preparation. However, the number of races per preparation remained constant, as did the spell length between preparations, indicating the pattern of racing remained the same for these horses. This demonstrates that within the current New Zealand system of racing and training horses, there was an opportunity for horses to have a longer racing career, but the biological constraints restricted both the length of each preparation and how often these horses raced within a preparation.

The number of horses and race opportunities in New Zealand has been decreasing since the 2008/9 racing season. This decrease was associated with a concurrent shift in the age profile of the horses racing, with an increasingly larger proportion of older horses racing in recent years. Thus, older horses are contributing disproportionately to provide the additional race starts to maintain industry equilibrium. This increasing ageing population of (predominantly) geldings may change the industry injury risk profile for the racing Thoroughbreds in New Zealand. Many injuries in racing are related to accumulation of cyclic load. Older horses will have accumulated greater cyclic load over their career and some tissues or structures, such as the superficial digital flexor tendon may be at greater risk of injury [24].

The shift in age profile appears to be a direct compensation for the decreasing number of horses entering the racing system. The decreasing number of horses entering the racing system has been in part a reflection of the lack of reinvestment in new racing product (new horses to enter racing), and a reduction in the supply chain, with a 20% reduction in the broodmare population between 2005/6–2015/16 [25]. The majority of the reduction in broodmares were those primarily generating horses for the domestic racing industry rather than the export market.

The proportion of young female horses racing in New Zealand is increasing. This may in part be due to the introduction of revised regulations in 2008 and 2011 where a 2 kg weight allowance in weight for age and open sex races was made for mares and fillies [26], giving them an added advantage, racing with lower weights than their male counterparts. Additionally, the increasing export focus of the New Zealand Thoroughbred breeding industry, with colts more marketable than fillies [25] may leave more fillies to compete in the domestic racing, with the colts sold to export [27]. With increasing age, there are fewer fillies/mares racing, reflecting the subsequent repurposing of racing mares as broodmares, with a mean age of mares entering breeding of 5 ± 2 years [28]. These fillies act in part to maintain industry equilibrium, with a higher proportion racing in recent years for an extra preparation before their retirement to stud. This new generation of mares may require a superior racing record to justify breeding than in previous years. 

Flat races in New Zealand were only offered within a limited range of distances. This may reflect horses specialising early at a narrow distance range. Horses in this study had little oscillation around the industry median flat race distance of 1400 m (IQR 1200 m–1670 m) [29]. If a change occurred in a horses’ race distance, it was early in their career. This may either reflect early individual distance specialisation or the alignment of racing product to meet the industry provision. Younger horses were more likely to race in the shorter distance races, with older horses dominating the longer distances. If changed, the race distance for a horse was likely to increase rather than decrease.

Spell length of racing horses decreased over the thirteen seasons, possibly in response to the falling number of horses entering racing. Resting horses during and between their racing and training campaigns is an important component of optimising performance and minimising injury risk. However, resting may also increase the risk of injury when horses return to training due to the resultant porosity of their bone [30,31]. The optimal frequency or duration of rest within or between racing preparations has not yet been determined. Younger horses in this study had a longer time between races within a training prep, but shorter spell lengths between preps. This may reflect the lower training volumes associated with 2-year-old horses [12] and thus the perception of reduced time required resting/spelling in association with the greater instructional training required for 2-year-olds rather than the ability to just focus on exercise capacity in older horses.

It has been previously reported that Thoroughbreds in New Zealand completed 2.5 starts per 100 training days, with a median of 17 days between successive starts for the same horse [32], similar to the pattern observed in this study. Horses begin a training preparation with slow work and progressively advance to a first start within 68 days after the start of a training preparation [32]. These training days would be included in the spell length calculations when spell length is estimated from race dates. Therefore, the actual days not training would be approximately 93 days (13 weeks), twice that observed in a study of Australian race horses, where horses 3 years and older were rested twice yearly for 6.3 weeks (44 days) with more experienced trainers resting their horses for shorter periods [12]. Two Australian studies have reported that age and targeted race distance were both associated with training workload, with 2-year-olds having longer spells than older horses [33,34]. This was in contrast to the pattern observed with the New Zealand data where 2-year-old horses had longer breaks between races and shorter spell lengths than older horses.

Spell frequency and duration depend on trainer and horse factors, but also climate, racing season and availability of spelling facilities. It was not possible with this dataset to distinguish between voluntary and involuntary (due to injury) spells. It has previously been reported in New Zealand that horses spent approximately 30% of their time resting, but fewer than 50% of rest periods were voluntary [32]. Therefore, both biological and economic drivers interact to affect the decreasing spell lengths for New Zealand Thoroughbreds.

The number of horses who had only one racing start in their career remained constant at 14% of the horses involved in racing. This may indicate that for a specific cohort, there are comparatively limited opportunities for these horses to race successfully in New Zealand. However, the consistency of this metric despite the falling number of race opportunities and horses competitively racing may point to early identification of those horses that are unable to sustain the training load required to compete successfully. Reasons for this may be injury predisposition, temperament or lack of talent [3], and allows these horses the option of early repurposing in other disciplines [35]. Early ejection of these horses from the industry may be a pragmatic approach by industry participants to maintain only those horses fit for racing, resulting in both welfare benefits for the horses as well as economic benefits to industry participants.

Balancing the system efficiency of the Thoroughbred racing industry in New Zealand requires both knowledge of horse welfare and physiology as well as an overview of current practices of the industry as a whole. A whole systems approach can then be used to determine the optimum number of horses required to maintain a functional level of racing. Even so, such a model may not account for the geographical distribution of horses and race days, or the possibility of injury to horses racing and subsequent increase in spell length (loss of racing days).

To maintain the optimum number of horses in a race (starters) to provide sufficient economic return, the industry either needs to maintain the current number of horses entering racing, or the decreasing number of horses that enter racing could compete in more races. Currently, the industry appears to be compensating for a falling number of horses by both keeping geldings in racing for longer and giving fillies more races before retirement, hence the ageing population of racehorses. Increasing the number of races these horses are able to compete in by optimising their training schedules would provide ample extra racing options. However, this would change their injury risk profile and may require a tighter knowledge of injury predisposition factors in racehorses. Further investigation on the metrics and causes for musculoskeletal injury in training and racing horses in longitudinal studies in different jurisdictions may inform future injury risk profiles.

## 5. Conclusions

The number of horses and races in the New Zealand Thoroughbred industry is decreasing. In association with this, there is an increasing proportion of 2–4-year-old female horses racing. To maintain the industry equilibrium, there has been an increasingly ageing population of active (gelding) racehorses, with horses more likely to be kept in racing for an extra preparation. This indicates that starts per preparation and length of preparation may be biologically constrained despite economic drivers for an increase. A consistent loss of 14% of new entrants each season indicates early identification of individuals that are unable to sustain the training load early and thus minimises injury risk in the racing population, increasing horse welfare.

## Figures and Tables

**Table 1:** Physical characteristics of racing starts and spells in Thoroughbred flat racing in New Zealand during the 2005/6 and 2017/8 racing seasons.

Racing Season	Total Starts	Starters	Starters per Race	New Entrants	Horses with One Start	Starts by Horses ≥ 6 Year-Old	Simpsons Index
	*n*		Median (IQR)	*n* (%)			(Age Profile)
2005/6	29,751	5559	11 (9–13)	-	824 (15%)	5388 (18%)	0.30
2006/7	30,574	5489	11 (9–14)	2250 (41%)	793 (14%)	6099 (20%)	0.32
2007/8	31,276	5512	11 (9–13)	2131 (39%)	819 (15%)	6832 (22%)	0.34
2008/9	33,061	5776	11 (9–14)	2315 (40%)	804 (14%)	7606 (23%)	0.35
2009/10	32,349	5732	11 (9–13)	2218 (39%)	823 (14%)	7214 (22%)	0.35
2010/11	31,605	5650	11 (9–13)	2174 (38%)	810 (14%)	7364 (23%)	0.36
2011/12	31,488	5562	11 (9–13)	2194 (39%)	736 (13%)	6939 (22%)	0.34
2012/13	30,380	5422	10 (9–12)	2081 (38%)	766 (14%)	6601 (22%)	0.34
2013/14	29,160	5333	10 (8–12)	2014 (38%)	755 (14%)	6882 (24%)	0.36
2014/15	28,872	5263	10 (8–12)	1962 (37%)	732 (14%)	7198 (25%)	0.37
2015/16	28,708	5119	11 (9–13)	1881 (37%)	687 (13%)	7307 (25%)	0.38
2016/17	25,982	4815	11 (8–13)	1726 (36%)	679 (14%)	6338 (24%)	0.37
2017/18	25,758	4691	10 (8–12)	1749 (37%)	648 (14%)	6166 (24%)	0.36

**Table 2:** Biological characteristics of racing starts and spells in Thoroughbred flat racing in New Zealand during the 2005/6 and 2017/8 racing seasons.

Racing Season	Starts per Horse	Races within a Prep	Days between Races within Prep	Number of Preps	Prep Length (Days)	Spell Length (Days)
	Median (IQR)					
2005/6	4 (2–8)	3 (2–5)	16 (12–22)	2 (2–2)	56 (28–94)	125 (84–182)
2006/7	5 (2–8)	3 (2–6)	16 (12–22)	2 (2–2)	63 (28–116)	169 (112–234)
2007/8	5 (2–8)	3 (2–6)	16 (12–22)	2 (2–2)	64 (28–116)	168 (108–238)
2008/9	5 (2–8)	3 (2–6)	16 (12–22)	2 (2–2)	65 (28–115)	166 (109–236)
2009/10	5 (2–8)	3 (2–5)	16 (12–23)	2 (2–2)	65 (28–120)	167 (109–237)
2010/11	5 (2–8)	3 (2–5)	16 (12–23)	2 (2–2)	66 (28–119)	165 (105–231)
2011/12	5 (2–8)	3 (2–6)	17 (12–23)	2 (2–2)	65 (28–119)	163 (106–231)
2012/13	5 (2–8)	3 (2–5)	17 (13–23)	2 (2–3)	65 (28–119)	163 (105–228)
2013/14	5 (2–8)	3 (2–5)	17 (13–24)	2 (2–2)	64 (28–119)	160 (105–225)
2014/15	5 (2–8)	3 (2–5)	18 (13–24)	2 (2–2)	65 (28–118)	163 (106–231)
2015/16	5 (2–8)	3 (2–5)	17 (13–24)	2 (2–3)	66 (28–120)	161 (105–223)
2016/17	4 (2–8)	3 (2–5)	18 (14–26)	2 (2–3)	66 (28–119)	157 (103–224)
2017/18	5 (2–8)	3 (2–5)	18 (14–24)	2 (2–2)	66 (28–114)	160 (105–223)

**Table 3:** Number of racing starts, days between races within a prep and spell length for horses of different ages for the 2005/6–2018/18 racing seasons in New Zealand.

Horse Age (Years)	Number of Racing Starts *n* (%)		Starts per Horse	Days between Races within Prep	Spell Length (Days)	Race Distance (m)
			Median (IQR)			
2	12,241	(3%)	2 (1–3)	21 (14–28)	114 (82–154)	1200 (1000–1200)
3	86,442	(22%)	4 (2–6)	18 (14–24)	139 (95–187)	1400 (1200–1600)
4	114,603	(29%)	5 (3–9)	17 (13–23)	167 (110–229)	1400 (1200–1600)
5	87,744	(23%)	6 (3–10)	16 (12–23)	168 (109–237)	1600 (1400–2000)
≥6	87,934	(23%)	7 (3–14)	15 (11–22)	181 (112–261)	1600 (1400–2000)

## Data Availability

Data are available in a public, open access repository. All data used in this study is freely available in a non-collated format on the website of New Zealand Thoroughbred Racing Inc. (www.nzracing.co.nz, accessed on 1 June 2019).
